# Vitamin C effects on 5-hydroxymethylcytosine and gene expression in osteoblasts and chondrocytes: Potential involvement of PHD2

**DOI:** 10.1371/journal.pone.0220653

**Published:** 2019-08-07

**Authors:** Richard C. Lindsey, Shaohong Cheng, Subburaman Mohan

**Affiliations:** 1 Musculoskeletal Disease Center, VA Loma Linda Healthcare System, Loma Linda, CA, United States of America; 2 Center for Health Disparities and Molecular Medicine, School of Medicine, Loma Linda University, Loma Linda, CA, United States of America; 3 Division of Biochemistry, Department of Basic Sciences, School of Medicine, Loma Linda University, Loma Linda, CA, United States of America; 4 Department of Medicine, School of Medicine, Loma Linda University, Loma Linda, CA, United States of America; 5 Department of Orthopedics, School of Medicine, Loma Linda University, Loma Linda, CA, United States of America; Kyungpook National University School of Medicine, REPUBLIC OF KOREA

## Abstract

Vitamin C (ascorbic acid, AA) is a well-known regulator of bone and cartilage metabolism. However, the mechanisms of AA’s action in these tissues are only partly understood. In this study, we confirmed that AA contributes to bone and cartilage metabolism by showing decreased articular cartilage and trabecular bone in AA-deficient spontaneous fracture (*sfx*) mutant mice. *In vitro*, we found that AA exerts differential effects on chondrocyte and osteoblast differentiation. Since AA is known to increase levels of 5-hydroxymethylcytosine (5-hmC) and induce DNA demethylation via the ten-eleven translocases (TETs), and since prolyl hydroxylase domain-containing protein 2 (PHD2), a known mediator of AA’s effects in these tissues, is part of the same enzyme family as the TETs, we next investigated whether increases in 5-hmC might mediate some of these effects. All TETs and PHDs are expressed in chondrocytes and osteoblasts, and PHD2 is localized in both the cytoplasm and nucleus of the cell, lending plausibility to the hypothesis of altered 5-hmC content in these cells. We found that AA treatment increased levels of 5-hmC in both cell types globally, notably including promoter regions of osteoblast differentiation genes. Furthermore, inhibition of PHD2 decreased 5-hmC levels in chondrocyte differentiation gene promoters, and knockdown of *Phd2* in chondrocytes reduced global 5-hmC levels, suggesting for the first time that PHD2 may itself directly mediate increases in 5-hmC in chondrocyte and osteoblast genes. Further investigation of this mechanism could lead to novel therapeutic approaches to treat debilitating diseases such as osteoarthritis and osteoporosis.

## Introduction

Vitamin C (ascorbic acid, AA) is a known regulator of both bone and cartilage metabolism. Epidemiological evidence suggests links between vitamin C deficiency and risk of osteoporosis and osteoarthritis. For example, a recent systematic review and meta-analysis of observational studies found that greater dietary vitamin C intake was associated with decreased risks of hip fracture and osteoporosis and higher bone mineral density (BMD) at the femoral neck and lumbar spine, [1] and a case-control study showed that vitamin C is a possible risk factor for osteoarthritis in the knee. [2] Additionally, many studies have confirmed a key role for vitamin C in the regulation of osteoblasts [3–11] and chondrocytes. [12–14]

The importance of vitamin C for musculoskeletal tissues has been confirmed in animal models. We and others have previously published studies of AA-deficient rodents which had impaired osteoblast differentiation, reduced bone formation, and development of spontaneous fractures. [15–22] Studies on the mechanisms by which AA regulates gene expression in bone cells have identified both direct and indirect routes of vitamin C action. [23] For example, vitamin C is an antioxidant, and AA treatment of bone marrow stromal cells increases expression of osteoblast differentiation regulator osterix (*Osx*) by causing nuclear factor-E2-related factor-1 (Nrf1) to bind an antioxidant-responsive element (ARE) in the *Osx* promoter. [24] Another mechanism by which AA regulates gene expression is via its role as a cofactor of prolyl hydroxylase domain-containing protein 2 (PHD2). Our previous studies have shown that many of the skeletal and cartilage effects of AA are mediated via PHD2. [25] The most well studied mechanism of PHD2 involves the hypoxia signaling pathway. PHD2 hydroxylates HIF1α, thereby marking it for ubiquitin-mediated proteasomal degradation. In the absence of oxygen, PHD2 cannot function, leading to accumulation of HIF1α to transcriptionally regulate its downstream target genes. We have previously shown that proteasomal degradation is important for the ability of AA to induce osterix (*Osx*) expression in osteoblasts. [26] However, this mechanism may not explain all the effects of AA in bone and cartilage.

Another possible mechanism for AA regulation of gene expression involves DNA methylation status. When a gene’s promoter is enriched in methylated cytosine (5-methylcytosine, 5-mC), transcription of that gene is repressed. The ten-eleven translocation (TET) hydroxylase enzymes demethylate DNA; through a series of oxidation reactions, the 5-mC is converted to 5-hydroxymethylcytosine (5-hmC) and 5-formylcytosine (5-fC) and is eventually replaced by an unaltered cytosine, thus allowing the gene to be transcribed. The TETs are part of a family of 2-oxoglutarate- and iron-dependent dioxygenases which require AA to function. Indeed, AA has been shown to induce DNA demethylation via the TETs in embryonic stem cells and some cancers. [27–29] Furthermore, changes in 5-hmC levels have been observed in osteoarthritic human chondrocytes, [30] and CpG islands in promoter regions of chondrocyte differentiation genes were consistently hypomethylated in human synovium-derived mesenchymal stem cells, [31] suggesting that regulation of DNA demethylation may be important for musculoskeletal function.

Interestingly, the PHDs are part of the same enzyme family as the TETs, and a recent study has provided evidence that, in addition to its role as a reducing agent, L-ascorbate acts as a true co-substrate for PHD2 in the same way it does for the TETs. [32] Since AA can induce DNA demethylation, and since PHD2 mediates AA effects in bone and cartilage, we hypothesized that some of the AA effect in osteoblasts and chondrocytes may be due to TET- and/or PHD2-mediated increases in 5-hmC in the promoter regions of genes important for osteoblast and chondrocyte function. To test this hypothesis, we first examined the effects of AA on bone and cartilage at a phenotypic and gene expression level. Next, we measured levels of 5-hmC in osteoblasts and chondrocytes after AA treatment. Finally, we determined the levels of 5-hmC in the promoter regions of osteoblast and chondrocyte genes after AA treatment.

## Materials and methods

### Mice

In order to investigate the effect of AA deficiency in mice *in vivo*, we used mice which have a deletion in the *GULO* gene which encodes gulonolactone oxidase, a key enzyme in the AA synthesis pathway, and are thus AA-deficient. Since these mice develop spontaneous fractures, they are known as *sfx* mice. [18] AA-deficient *sfx* mice and their heterozygous, AA-replete littermates were fed an AA-free diet before skeletal and articular cartilage phenotype analysis via microCT and histology, respectively. In our previous studies, we have determined that there were no significant differences either in serum vitamin C levels or skeletal phenotype between heterozygous *sfx*/+ littermates and wild type littermates. Thus, using heterozygous littermates as controls allows us to use a breeding scheme (*sfx*/+ X *sfx*/*sfx*) which increases the yield of mutant mice to allow for higher sample sizes in experiments. Additionally, primary bone and cartilage cells were isolated from wild-type C57BL/6 mice and *Phd2*-floxed mice and grown in culture for *in vitro* experiments. Mice were housed at the VA Loma Linda Healthcare System Veterinary Medical Unit (Loma Linda, CA, USA) under standard approved laboratory conditions. All the procedures were performed with the approval of the Institutional Animal Care and Use Committee of the VA Loma Linda Healthcare System. For euthanasia, animals were exposed to CO_2_ prior to cervical dislocation.

### Micro-computed tomography

The trabecular bone phenotype of 8-week-old *sfx* mice and their control littermates was evaluated at the primary and secondary femoral spongiosa via micro-computed tomography (microCT, Scanco vivaCT 40, Scanco Medical, Bruttisellen, Switzerland). The bones were scanned at a resolution of 10.5 μm with a 55kVp X-ray for measurement of trabecular bone microstructure. For primary spongiosa, a region of 0.36 mm starting from the end of the growth plate of the proximal femur was analyzed. For secondary spongiosa, a 1.2 mm region starting 0.36 mm distal to the growth plate was analyzed. The exact numbers and location of the slices used for analysis were corrected for bone length such that the analyzed regions were anatomically comparable between samples. Trabecular bone parameters were analyzed as described previously. [25]

### Histology

The articular cartilage phenotype of 8-week-old *sfx* mice and their control littermates was evaluated at the femoral and tibial aspects of the knee joint. The knee joints were decalcified in 20% EDTA at 4°C for 4 weeks, dehydrated using alcohol, and embedded in paraffin. The paraffin-embedded knee joints were sectioned at a thickness of approximately 4 μm, and the sections were stained with Safranin-O using standard protocols. Articular cartilage area and width were quantitated (18–30 sections per knee joint) by a blinded observer using the OsteoMeasure system (OsteoMetrics, Atlanta, GA, USA). OARSI scoring was conducted as previously described. [33, 34]

### Cell culture

Primary mouse calvarial osteoblasts and rib, growth plate, or epiphyseal chondrocytes were isolated from C57BL/6 mice or *Phd2*-floxed mice via enzymatic digestion with collagenase I (2 mg/mL) and hyaluronidase (1 mg/mL) or collagenase D (2 mg/mL), respectively. Primary cells and the ATDC5 (Abgent, San Diego, CA, USA) and MC3T3-E1 (ATCC, Manassas, VA, USA) cell lines were grown in AA-free alpha-MEM or DMEM/F-12 growth medium supplemented with 10% fetal bovine serum, penicillin (100 units/mL), and streptomycin (100 μg/mL) to 80% confluence before beginning experiments. ATDC5 cells were grown to confluence and treated with ascorbic acid and β-glycerophosphate; they were not induced to differentiate with, e.g., insulin and dexamethasone. Cells from *Phd2*-floxed mice were transduced according to previously published procedures [35] with adenoviral GFP or iCre vectors (produced by our colleague Weirong Xing) to knock down expression of *Phd2*. Knockdown was measured via real-time RT-PCR as described below. For each assay described below, cells were synchronized by serum starvation for 24 hours before 3-day treatment with 50 μg/mL (284 μM) AA and 10 mM β-glycerophosphate (unless other treatment durations and concentrations are specified). Chemical inhibition of PHD2 was achieved by treatment with PHD2-specific inhibitor Iox2 (20 μM; Cayman Chemical, Ann Arbor, MI, USA). To make sure that the effects of AA treatment are due to the AA we added as a treatment, all experiments were conducted in growth media which contained no AA.

### Cell proliferation assay

Cells were plated at a density of 3,000 cells/well in a 96 well plate. One day later, a 24 hour period of serum-free synchronization was begun, followed by addition of media containing AA (50 μg/mL; 284 μM) and 10 mM β-glycerophosphate. Proliferation was measured 48 hours later using the CyQUANT kit according to the manufacturer’s instructions (ThermoFisher Scientific, Waltham, MA, USA).

### Alkaline phosphatase (ALP) activity assay

Briefly, cells were plated at a density of 5,000 cells/well in a 96 well plate and allowed to proliferate for 24 hours before a 24 hour period of serum-free synchronization followed by addition of AA (doses ranging from 0–250 μg/mL, 0–1.42 mM) with 10 mM β-glycerophosphate. After 72 hours of treatment, cells were permeabilized with Triton X-100, PNPP substrate was added, and absorbance was read in a plate reader at intervals 0, 1, 3, and 5 hour time points. The difference in absorbance at 490 nm and 410 nm was used to calculate enzyme activity over time, which was normalized to total protein concentration as measured by a Pierce^™^ BCA kit according to manufacturer’s instructions (ThermoFisher Scientific, Waltham, MA, USA).

### RNA extraction and quantitation

RNA samples were extracted from primary mouse osteoblasts and chondrocytes as well as the ATDC5 and MC3T3-E1 cell lines with TRI Reagent (MRC, Cincinnati, OH, USA) and the E.Z.N.A.^®^ Total RNA Kit I (Omega Bio-tek, Norcross, GA, USA) according to the manufacturers’ instructions. An aliquot of RNA (300 ng) was reverse-transcribed using SuperScript II^®^ reverse transcriptase (Invitrogen, Grand Island, NY, USA). Quantitative real-time RT-PCR was performed as previously described. The DDCT method was used to calculate relative gene expression with the housekeeping gene PPIA serving as an internal control as previously described. [36] Primer sequences can be found in Table 1.

**Table 1:** Primer sequences for real-time PCR.

Gene	Forward	Reverse
Aggrecan	5′-GACCAGGAAGGGAGGAGTAG-3′	5′-CAGCCGAGAAATGACACC-3′
ALP	5′-ATGGTAACGGGCCTGGCTACA-3′	5′-AGTTCTGCTCATGGACGCCGT-3′
BiP	5′-TTCAGCCAATTATCAGCAAACTCT-3′	5′-TTTTCTGATGTATCCTCTTCACCAGT-3′
BSP	5′-CTCTACTCCGTCTGCACAACA-3′	5′-TAAGCTCGGTAAGTGTCGCCA-3′
CHOP	5′-CCACCACACCTGAAAGCAGAA-3′	5′-AGGTGAAAGGCAGGGACTCA-3′
Col10	5′-ACGGCACGCCTACGATGT-3′	5′-CCATGATTGCACTCCCTGAA-3′
Col2	5′-TGGCTTCCACTTCAGCTATG-3′	5′-AGGTAGGCGATGCTGTTCTT-3′
Gpx2	5′-ACCGATCCCAAGCTCATCAT-3′	5′-CAAAGTTCCAGGACACGTCTGA-3′
GSR	5′-GCTATGCAACATTCGCAGATG-3′	5′-AGCGGTAAACTTTTTCCCATTG-3′
Gsta2	5′-CGTCCACCTGCTGGAACTTC-3′	5′-GCCTTCAGCAGAGGGAAAGG-3′
Ihh	5′-CCCCAACTACAATCCCGACATC-3′	5′-CGCCAGCAGTCCATACTTATTTCG-3′
Jmjd1a	5′-CCAGGAGAAGACTTCAGAGACATG-3′	5′-GGTGTACTCAGGCAGTGGAATG-3′
Jmjd2b	5′-GGCCAAGATCATTCCACCCA-3′	5′-CCCACAGTCATGGCCTTCTT-3′
Osx	5′-TCCTCTCTGCTTGAGGAAGAAG-3′	5′-GAGTCCATTGGTGCTTGAGAAG-3′
Phd1	5′-GGAACCCACATGAGGTGAAG-3′	5′-AACACCTTTCTGTCCCGATG-3′
Phd2	5′-GAAGCTGGGCAACTACAGGA-3′	5′-CATGTCACGCATCTTCCATC-3′
Phd3	5′-AAGTTACACGGAGGGGTCCT-3′	5′-GGCTGGACTTCATGTGGATT-3′
PPIA	5′-CCATGGCAAATGCTGGACCA-3′	5′-TCCTGGACCCAAAACGCTCC-3′
Tet1	5′-AGGAAATGCGAGGTGCTCAA-3′	5′-TCCCCATGACCACGTCTACT-3′
Tet2	5′-AGCTCGAAAGCGTTCCTCTC-3′	5′-GAAGGTGCCTCTGGAGTGTT-3′
Tet3	5′-GCATGTACTTCAACGGCTGC-3′	5′-TCGCCACATCCTCATTGGTC-3′

### Protein extraction and quantitation

Cytoplasmic and nuclear protein samples were extracted from MC3T3-E1 pre-osteoblasts using a previously described method. [37] Briefly, cells were scraped, centrifuged and resuspended in PBS, and lysed with a buffer containing NP-40 on ice for 10 minutes. Nuclei were pelleted by centrifugation (2,500 RCF at 4°C for 10 minutes), and the supernatant containing cytosolic proteins was collected. Nuclei were washed and resuspended in a glycerol-containing buffer with 0.4 M NaCl followed by occasional vortexing at 4°C for 20 minutes. Following centrifugation (12,000 RCF at 4°C for 10 minutes), the supernatant containing nuclear proteins was collected. Protein samples were analyzed by immunoblotting of PVDF membranes with protein transferred from SDS-PAGE gels (antibodies used were as follows: EGLN1/PHD2, 48355, Cell Signaling, Danvers, MA, USA; β-actin, A1978, Sigma-Aldrich, St. Louis, MO, USA; histone 3, H0164, Sigma-Aldrich, St. Louis, MO, USA) followed by chemiluminescent imaging. Blots were quantitated using ImageJ.

### Immunofluorescence microscopy

ATDC5 pre-chondrocytes and primary mouse rib chondrocytes were plated at a density of 20,000 cells/well on 8-well Lab-Tek^™^ Chamber Slides^™^ (177402, Thermo Fisher Scientific, Waltham, MA, USA), allowed to proliferate for 24 hours, and synchronized by serum starvation before treatment with AA. After three of days of treatment, cells were washed with PBS, fixed with 4% paraformaldehyde at room temperature for 10 minutes, washed again with PBS, and permeabilized with 0.5% Triton X-100 in 1X PBS for five minutes. Permeabilization buffer was washed off with PBS before blocking with 2.5% normal horse serum (from the VectaFluor^™^ R.T.U. Anti-Rabbit IgG kit, Vector Laboratories, Burlingame, CA, USA) for 1 hour at room temperature. Cells were incubated with primary antibody (PHD2, Cell Signaling; 1:200) in 1% normal horse serum overnight at 4°C. The next day, cells were washed with PBS three times for five minutes each, incubated with fluorophore-conjugated secondary antibodies (ATDC5: VectaFluor^™^ R.T.U. DyLight^®^ 488 Anti-Rabbit IgG [DI-1788, Vector Laboratories]; rib chondrocytes: VectaFluor^™^ R.T.U. DyLight^®^ 594 Anti-Rabbit IgG [DI-1794, Vector Laboratories]) for one hour at room temperature, washed again with PBS, counterstained with DAPI (286 nM), and rinsed again with PBS. Slide chambers and gaskets were removed, and slides were mounted with Fluoromount-G^®^ (0100-01, SouthernBiotech, Birmingham, AL, USA). Cells were imaged using an Olympus Fluoview FV3000 confocal laser scanning microscope (Olumpus, Center Valley, PA, USA). Negative controls were processed in the same manner, excluding primary antibody in the primary antibody step. ATDC5 negative controls were processed under identical conditions but at a later time than the experimental samples.

### 5-hmC quantitation

After synchronization by serum starvation for 24 hours, chondrocytes and osteoblasts were treated with AA (50–100 μg/mL; 284–568 μM) for 3 days. DNA was extracted using the DNEasy Blood & Tissue DNA extraction kit (Qiagen, Germantown, MD, USA). 5-hmC was measured by dot blot as follows. Briefly, DNA was deposited on nitrocellulose membranes in 1 μL droplets containing a series of 2-fold dilutions beginning at 100 ng/μL, UV crosslinked to the membrane (120,000 μJ/cm^2^), dried at 80°C, rinsed with 2X saline-sodium citrate, and probed with a 1:1000 dilution of 5-hmC antibody (Diagenode, Denville, NJ, USA) for 1 hour before chemiluminescent imaging. Blots were quantitated using ImageJ. 5-hmC ELISA was performed using a kit (Epigentek, Farmingdale, NY, USA) according to manufacturer’s instructions.

### Quantitation of promoters enriched in 5-hmC

Extracted DNA (20 μg/sample) was diluted in 300 μL 10 mM Tris-HCl (pH 8.5) and sonicated with 15 pulses of 20 seconds at power level 2 (Sonic Dismembrator Model 100, Fisher Scientific, PA, USA), with a 20-second pause on ice between each pulse. The sheared DNA samples were fragmented to 100–500 bp in size and visualized by ethidium bromide staining after electrophoresis on a 2% agarose gel.

5-hmC was enriched using a kit according to manufacturer’s instructions (Hydroxymethyl Collector, Active Motif, Carlsbad, CA). Briefly, 2.5 μg of fragmented DNA was spiked with 5-hmC positive control DNA (human APC gene) with a ratio of 1 to 20,000. 10% of the spiked DNA samples were set aside as input. The DNA samples were glycosylated with the presence of 0.15 mM UDP-azide-glucose and 8 units of β-glucosyltransferase in 50 μL reaction volume at 37°C for 1 hour. A negative control reaction omitting UDP-azide-glucose was also set up for each DNA sample. The reaction was further incubated with 20 μL biotin conjugation solution at 37°C for 1 hour. The reactions were purified with the columns provided by the kit. The hydroxymethylated DNA fragments were then captured by streptavidin beads and further purified with provided columns.

CpG islands in promoter regions of the genes of interest were retrieved from the UCSC genome browser, and primer pairs were designed within the CpG islands with an average T_m_ of 60°C and PCR products of 100–200 bp. 5 μL of the 5-hmC enriched DNA were used to perform real-time PCR with a 20 μL SYBR Green reaction mix. Enrichment of 5-hmC DNA was analyzed with DDCT method, and the APC control primers were used for normalization.

### Statistical analysis

Results are expressed as mean ± SEM. Data were analyzed using Student’s *t*-tests or one-way ANOVA followed by pairwise *t*-tests with Bonferroni adjustments for multiple comparisons. Statistical software used were Microsoft Excel and R 3.5.3. [38].

## Results

### AA-deficient *sfx* mice had less articular cartilage

**Fig 1:**
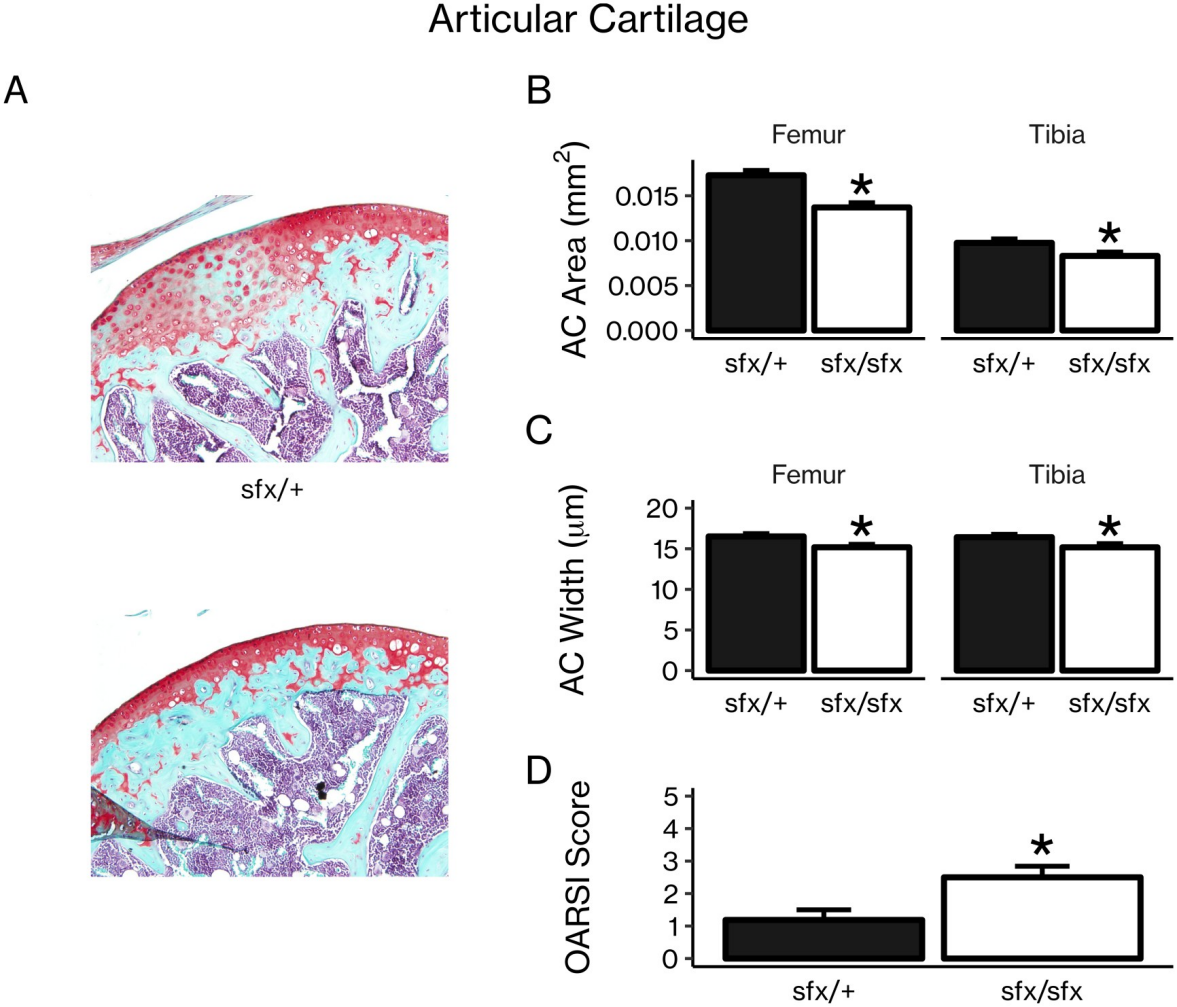
AA-deficient *sfx* mice had decreased articular cartilage. (A) Representative sections of articular cartilage from control *sfx*/+ mice and AA-deficient *sfx*/*sfx* mice stained with Safranin-O. Articular cartilage was quantitated using the OsteoMeasure software, and *sfx*/*sfx* mice had less articular cartilage area (B) and width (C) at the femur and tibia. OARSI scores (D) are shown for *sfx*/+ and *sfx*/*sfx* mice. Results are presented as mean ± SEM. *P < 0.05 vs. control (Student’s *t*-test), n = 6 per group.

To evaluate the importance of vitamin C in articular cartilage, we compared histologic slides of knee joints stained with Safranin-O from AA-deficient *sfx* mice and control littermates at 8 weeks of age (Fig 1A). At the knee joint, AA-deficient *sfx* mice had significantly decreased articular cartilage area (21% at the femoral aspect and 15% at the tibial aspect, Fig 1B). Similarly, both the femoral and tibial aspects of the knee joint had decreased articular cartilage width (8%, Fig 1C). These reductions in articular cartilage area and width are characteristics of an osteoarthritis-like phenotype, a finding further confirmed by OARSI scoring (Fig 1D).

### AA-deficient *sfx* mice had less trabecular bone

**Fig 2:**
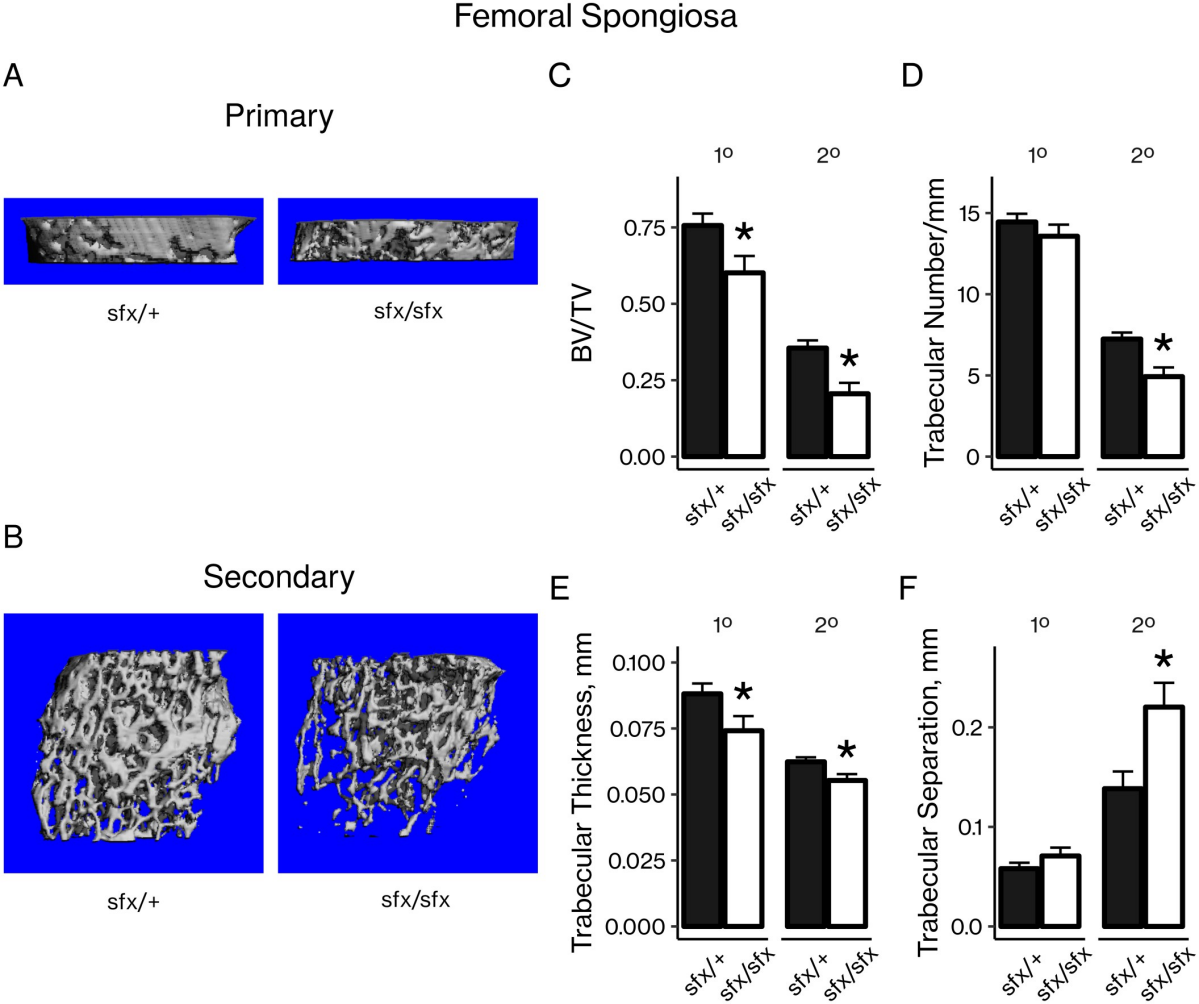
AA-deficient *sfx* mice had less trabecular bone at the femoral primary and secondary spongiosa. Representative microCT images of (A) primary and (B) secondary spongiosa from control *sfx*/+ mice and AA-deficient *sfx*/*sfx* mice. Scans were quantitated by (C) bone volume adjusted for tissue volume (BV/TV), (D) trabecular number, (E) trabecular thickness, and (F) trabecular separation. Results are presented as mean ± SEM. *P < 0.05 vs. control (Student’s *t*-test), n = 6 per group.

The primary and secondary spongiosa of 8-week-old AA-deficient *sfx* mice were analyzed by microCT (Fig 2A and 2B). AA-deficient mice had 20% and 42% less bone volume adjusted for tissue volume (BV/TV) at the primary and secondary spongiosa, respectively, compared to their control littermates (Fig 2C). These decreases were explained by a significant reduction in trabecular number at the secondary spongiosa (Fig 2D), significant reductions in trabecular thickness at both the primary and secondary spongiosa (Fig 2E), and a significant increase in trabecular separation at the secondary spongiosa (Fig 2F).

### AA had opposite effects on proliferation and differentiation in chondrocytes vs. osteoblasts

**Fig 3:**
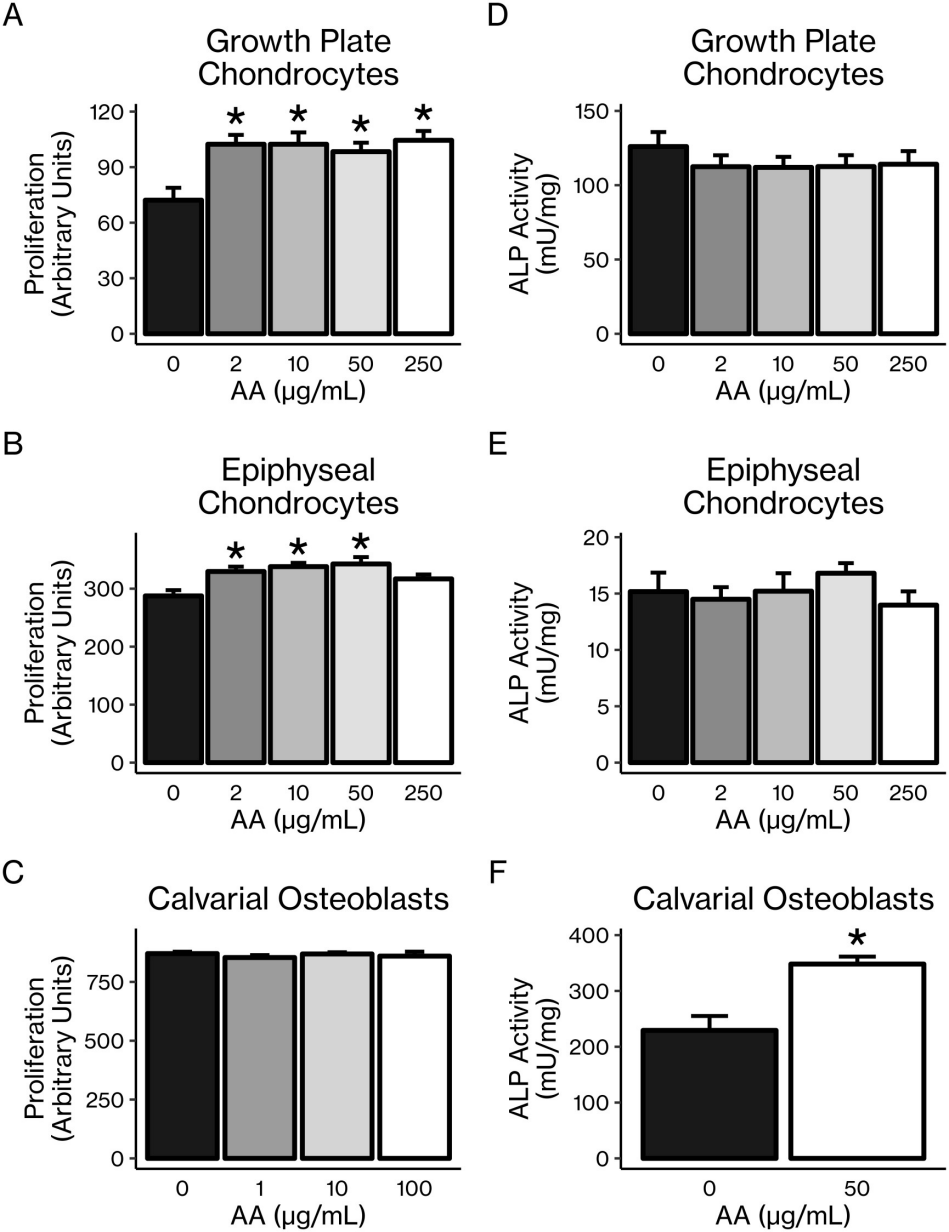
AA increased proliferation of primary chondrocytes and differentiation of osteoblasts *in vitro*. Proliferation of primary (A) growth plate chondrocytes and (B) epiphyseal chondrocytes was increased by AA treatment. Proliferation of (C) primary calvarial osteoblasts was unaffected by AA treatment. ALP activity of (D) growth plate chondrocytes and (E) epiphyseal chondrocytes was not changed by AA treatment, while ALP activity of (F) calvarial osteoblasts was increased by AA treatment. Results are presented as mean ± SEM. *P < 0.05 vs. control (pairwise *t*-tests with Bonferroni corrections for multiple comparisons [A–E] or Student’s *t*-test [F]), n = 6–8 per group.

Treatment of chondrocytes *in vitro* with AA led to significant 20–40% (*P* < 0.05) increases in cellular proliferation of primary growth plate and epiphyseal chondrocytes (Fig 3A and 3B), although this effect was not seen in the ATDC5 chondrocytic cell line under the specific conditions we observed (S1A Fig). By contrast, *in vitro* AA treatment had no effect on proliferation of primary calvarial osteoblasts (Fig 3C) or the pre-osteoblastic MC3T3 cell line (S1B Fig). However, the opposite was true of AA’s effect on ALP activity, a marker of differentiation. While AA treatment had no effect on ALP activity in either primary chondrocytes (Fig 3D and 3E) or the ATDC5 cell line under the conditions we observed (S1C Fig), ALP activity was significantly increased by 30–160% (*P* < 0.05) in both primary calvarial osteoblasts (Fig 3F) and the MC3T3-E1 cell line (S1D Fig).

### AA treatment regulated markers of osteoblast and chondrocyte differentiation and maturity

**Fig 4:**
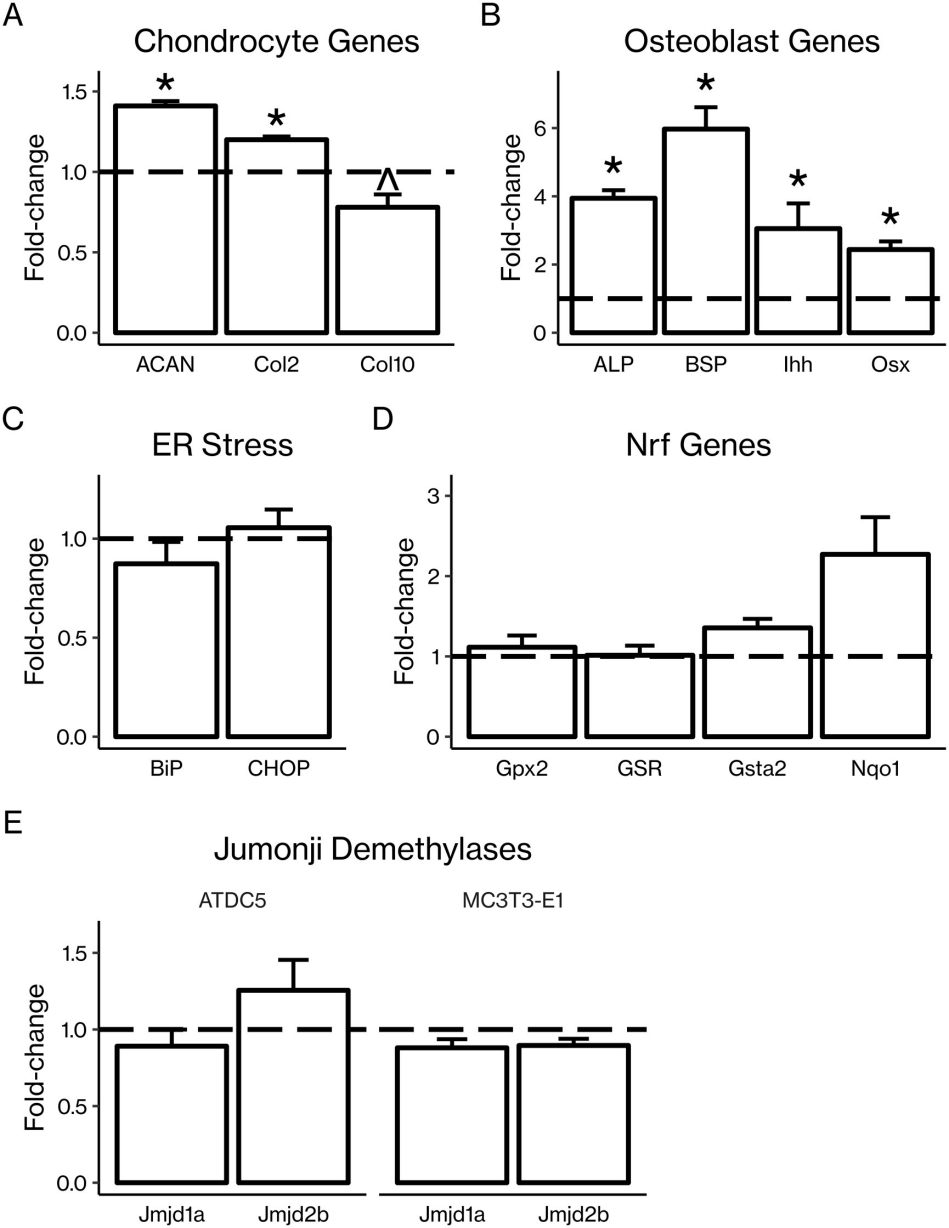
AA regulated expression of chondrocyte and osteoblast genes at the mRNA level. (A) Primary mouse articular chondrocytes were treated with AA (50 μg/mL) before RNA extraction and RT-qPCR. AA treatment significantly increased expression of immature chondrocyte genes aggrecan (AGCN) and type 2 collagen (Col2) but not mature chondrocyte differentiation marker type 10 collagen (Col10). (B) Primary mouse calvarial osteoblasts were treated with AA (50 μg/mL) before RNA extraction and RT-qPCR. AA treatment significantly increased expression of osteoblast differentiation genes alkaline phosphatase (ALP), bone sialoprotein (BSP), Indian hedgehog (Ihh), and osterix (Osx). ATDC5 chondrocytes were treated with AA before RNA extraction RT-qPCR, and no changes in (C) ER stress markers BiP or CHOP or (D) Nrf2 responsive genes Gpx2, GSR, Gsta2, or Nqo1 were observed. (E) AA treatment did not regulate expression of Jumonji family demethylases. demethylases. ATDC5 chondrocytes and MC3T3-E1 pre-osteoblasts were treated with AA before RNA extraction RT-qPCR. No significant changes in Jmjd1a or Jmjd2b were found. Results are presented as mean fold-change ± SEM. *P < 0.05 vs. control, ^*P* = 0.06 (Student’s *t*-test), n = 3–4 per group.

In primary mouse articular chondrocytes, AA treatment increased mRNA expression of immature chondrocyte markers *Acan* and *Col2* 1.2–1.4-fold (*P* < 0.05) while tending to decrease expression of mature chondrocyte marker *Col10* 0.8-fold (*P* < 0.1; Fig 4A). Consistent with the earlier finding that AA increased ALP activity in osteoblastic cells, we found that AA treatment in primary mouse calvarial osteoblasts produced 2.5–6-fold increases (*P* < 0.05) in mRNA expression of several markers of osteoblast differentiation including *Alp*, *Bsp*, *Ihh*, and *Osx* (Fig 4B).

### Differential regulation of TET and PHD expression in chondrocytes and osteoblasts

**Fig 5:**
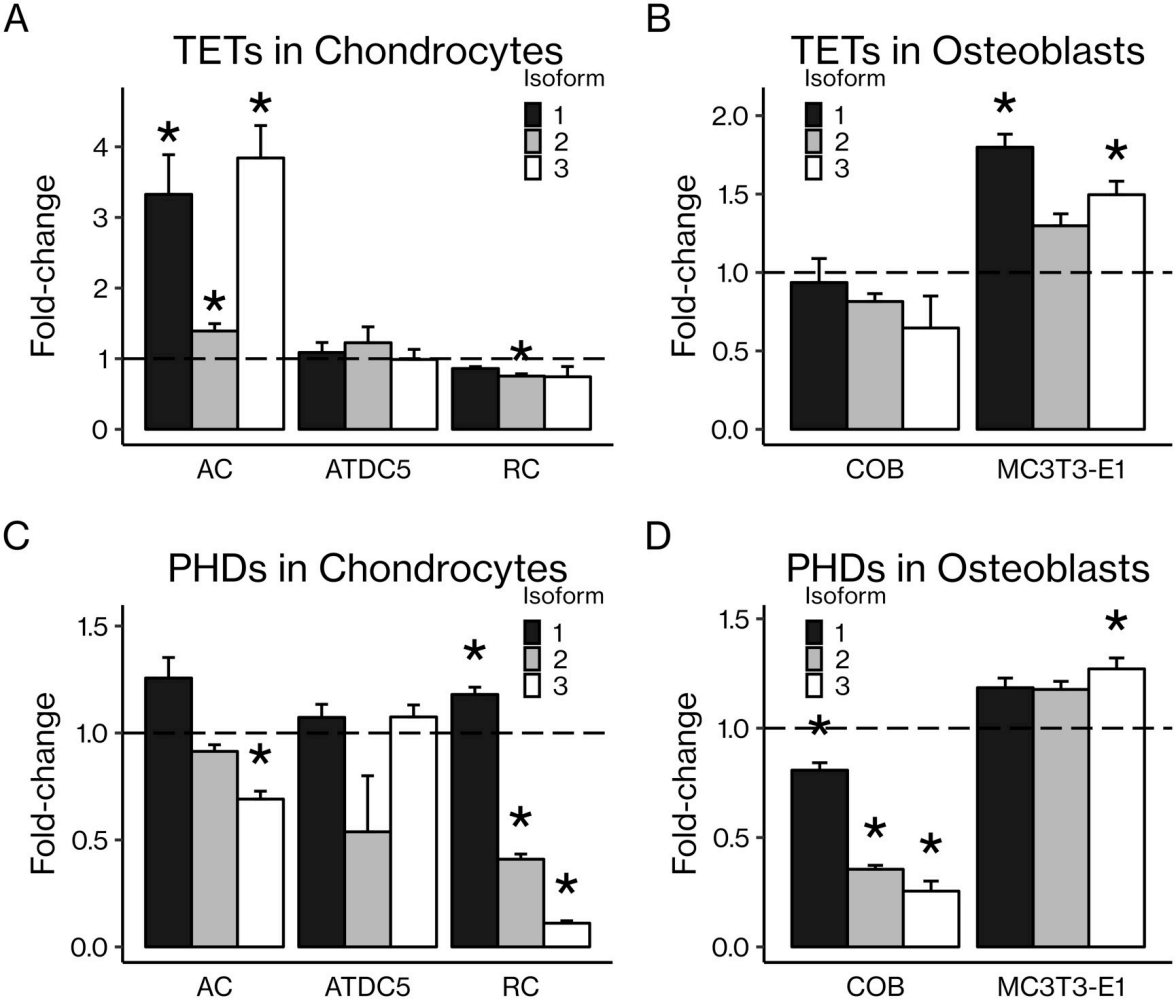
All TETs and PHDs are expressed in chondrocytes and osteoblasts at the mRNA level, and expression of many TETs and PHDs is regulated by AA. Primary articular chondrocytes (AC), ATDC5 chondrocytes, primary rib chondrocytes (RC), primary calvarial osteoblasts (COB), and MC3T3-E1 pre-osteoblasts were cultured *in vitro* and treated with AA (50 μg/mL) before RNA extraction and RT-qPCR. Expression of TETs in (A) chondrocytes and (B) osteoblasts as well as PHDs in (C) chondrocytes and (D) osteoblasts are reported. Results are presented as mean fold-change ± SEM. *P < 0.05 vs. control (Student’s *t*-test), n = 3–4 per group.

**Fig 6:**
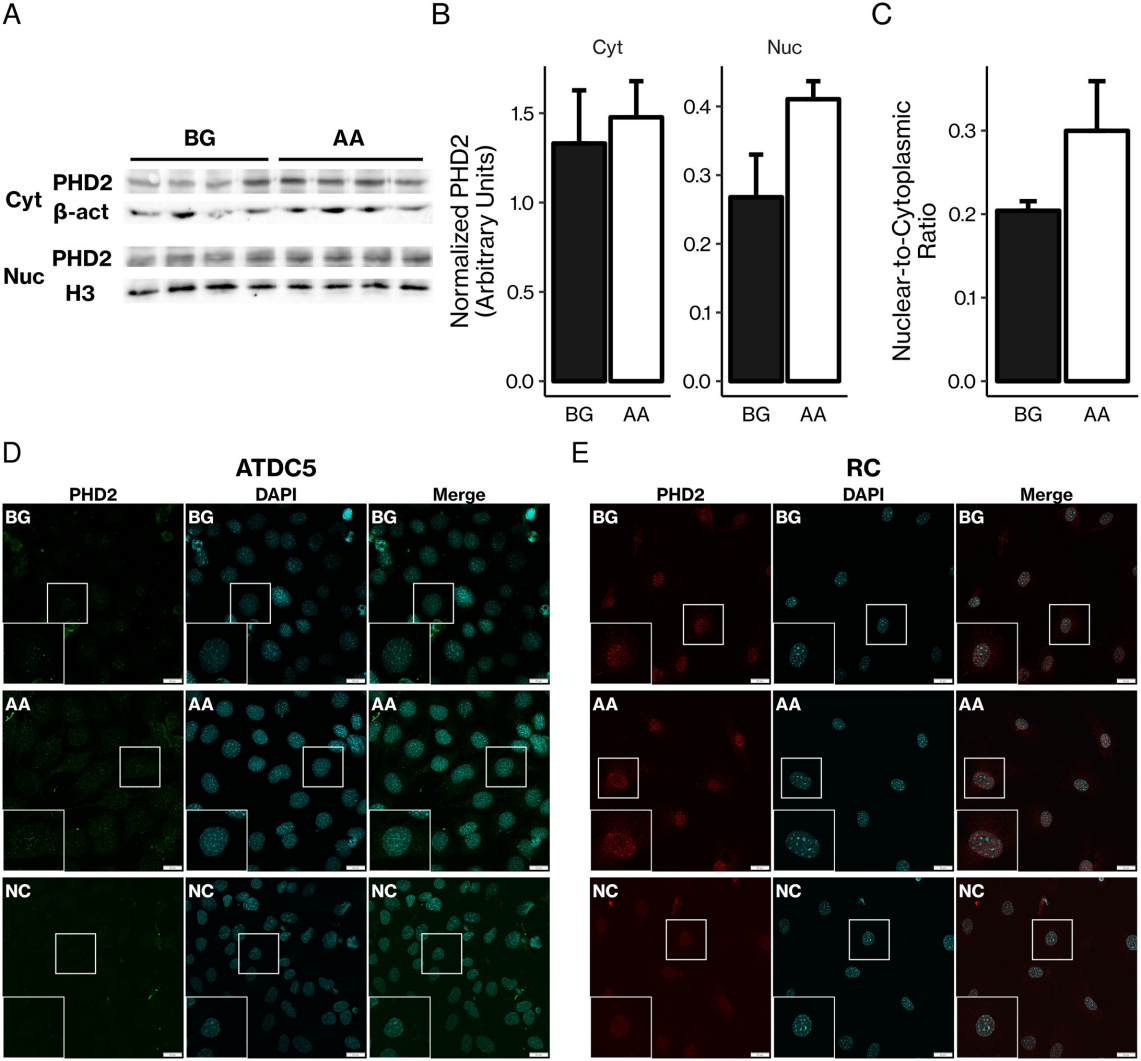
PHD2 is expressed in both the cytoplasm and nucleus at the protein level. (A) MC3T3-E1 pre-osteoblasts were treated with β-glycerophosphate (BG) with or without AA before cytoplasmic (Cyt) and nuclear (Nuc) protein extraction and SDS-PAGE followed by immunoblotting. Loading controls were β-actin (β-act) for the cytoplasmic fraction and histone 3 (H3) for the nuclear fraction. (B) Immunoblots were quantitated with ImageJ, and signals from each fraction and treatment group were normalized to the respective loading control signals to produce quantitated, normalized PHD2 levels in the cytoplasmic and nuclear fractions. (C) The nuclear fraction signal was divided by the cytoplasmic fraction signal to give an indirect measure of the ratio of PHD2 levels in the nucleus vs. cytoplasm. AA treatment did not significantly change the nuclear-to-cytoplasmic ratio (*P* = 0.2, Student’s *t*-test). Results are presented as mean ± SEM. n = 4 per group. (D) ATDC5 chondrocytes immunostained for PHD2. Inserts are magnifications of indicated regions. NC = negative control. Green = PHD2; blue = DAPI. (E) Rib chondrocytes immunostained for PHD2. Inserts are magnifications of indicated regions. NC = negative control. Red = PHD2; blue = DAPI. Scale bars = 20 μm.

All isoforms of TETs and PHDs were detected at the mRNA level by real-time PCR in primary and cell line chondrocytes and osteoblasts (Fig 5). Absolute mRNA levels are provided in S1 Table. *In vitro* AA treatment had varying effects on each isoform; TETs tended to increase with AA treatment while PHDs tended to decrease. For example, AA increased expression of all three TET isoforms in articular chondrocytes and in the MC3T3-E1 pre-osteoblastic cell line. AA decreased expression of *Phd3* in articular chondrocytes and all PHDs in calvarial osteoblasts. The varying effects of AA on TET and PHD mRNA expression appear to be dependent on cell type as well as stage of differentiation. Furthermore, PHD2 was expressed at the protein level in both the cytoplasm and nucleus of MC3T3-E1 calvarial osteoblasts, and AA treatment tended to increase the nuclear-to-cytoplasmic ratio of PHD2 expression (Fig 6A–6C), although this effect was not statistically significant. Additionally, immunofluorescent confocal micrographs of ATDC5 chondrocytes and rib chondrocytes stained for PHD2 show that PHD2 is present in both the cytoplasm and nucleus (Fig 6D and 6E).

### AA treatment did not regulate markers of ER stress or Nrf2 signaling

Vitamin C is known to modulate expression of antioxidant response element (ARE)-containing genes in part through the nuclear factor erythroid-derived 2-related factor (Nrf) proteins. [24, 39] In addition, crosstalk between Nrf2 and endoplasmic reticulum (ER) stress signaling has been established. [40] To determine whether AA’s effects may be mediated via regulation of ER stress or Nrf2 signaling, we measured expression of ER stress markers *BiP* and *CHOP* and Nrf responsive genes *Gpx2*, *GSR*, *Gsta2*, and *Nqo1* in ATDC5 chondrocytes after AA treatment, and we found no significant changes (Fig 4C and 4D).

### AA treatment increased 5-hmC levels in osteoblasts and chondrocytes

**Fig 7:**
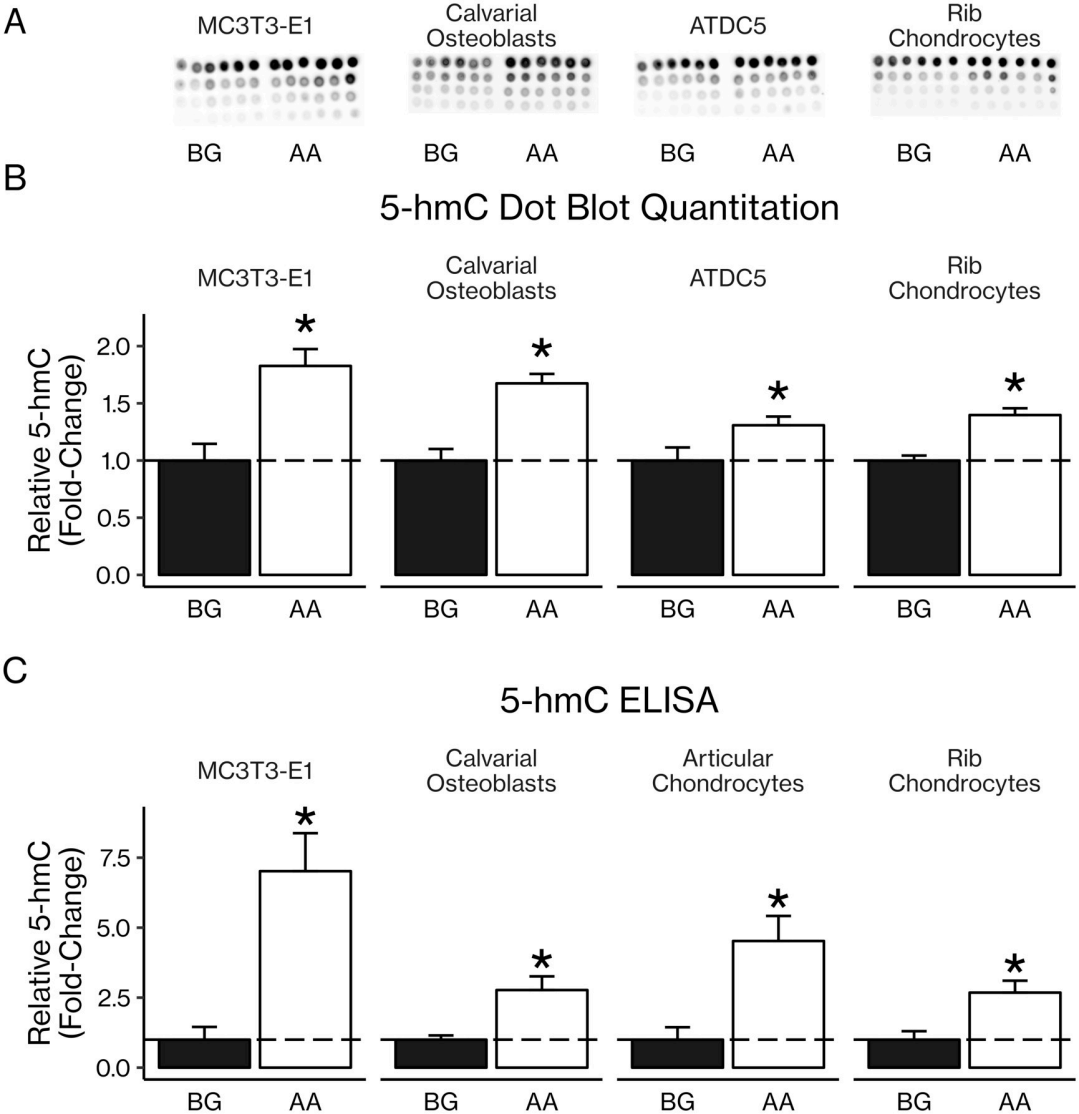
AA increased 5-hmC levels in osteoblasts and chondrocytes. (A) MC3T3-E1 pre-osteoblasts, primary calvarial osteoblasts, ATDC5 chondrocytes (not induced with, e.g., insulin and dexamethasone), and primary rib chondrocytes were treated with β-glycerophosphate (BG) with or without AA before DNA extraction and dot blotting using an antibody specific for 5-hmC. Each replicate is in a column on the dot blots, and each row indicates a serial two-fold dilution of the sample above it, beginning at 100 ng of DNA in each dot on the top row. (B) Dot blots were quantitated with ImageJ. (C) 5-hmC levels were also measured using an ELISA. Results are presented as mean fold-change ± SEM. *P < 0.05 (Student’s *t*-test), n = 6 per group for dot blot; technical issues during the ELISA led to several readings being invalidated, leaving the following n: MC3T3-E1 BG = 2, AA = 5; COB BG = 4, AA = 4; RC BG = 4, AA = 4; AC BG = 3, AA = 2.

**Fig 8:**
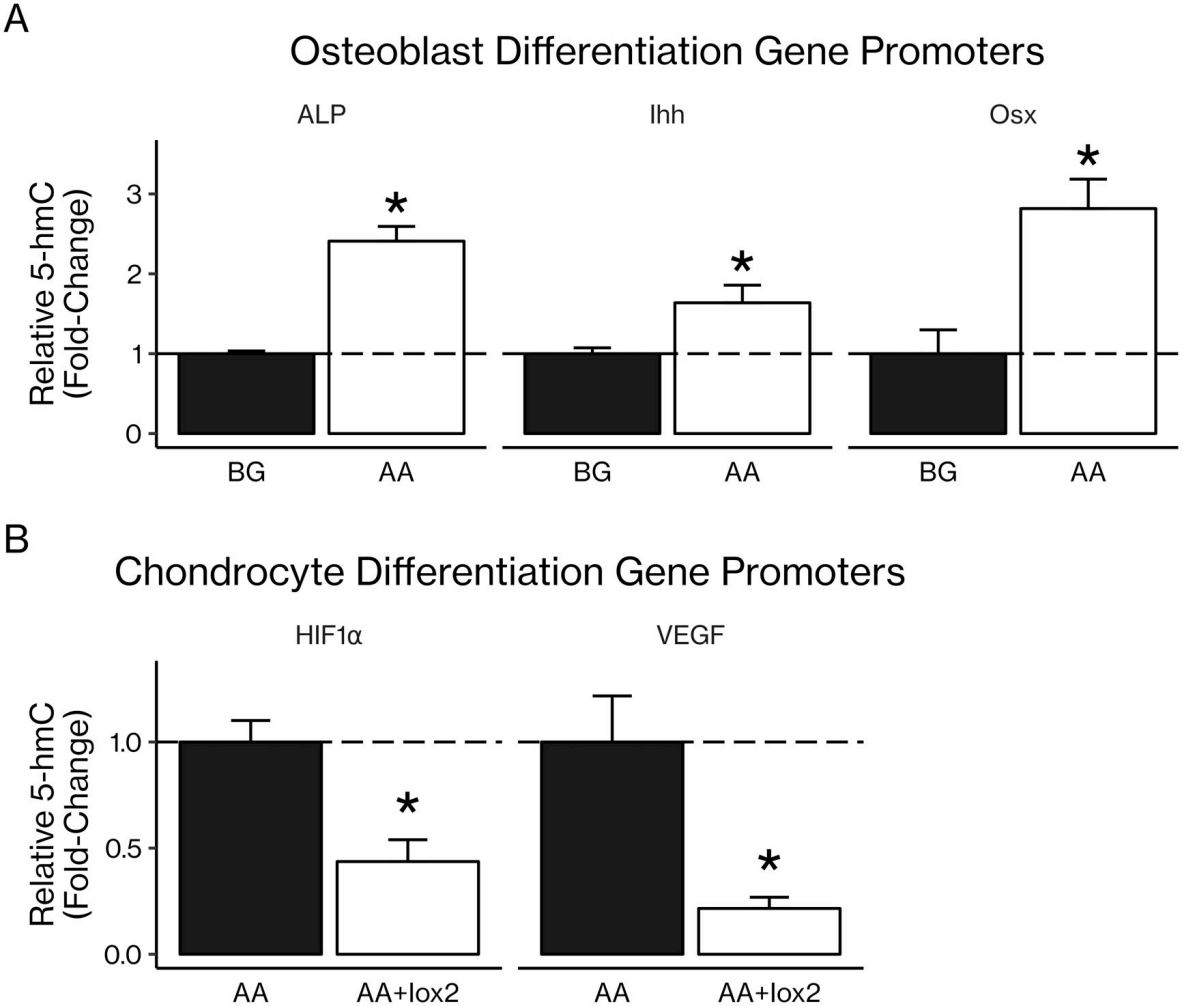
Increased 5-hmC occurs in osteoblast and chondrocyte gene promoters. After treatment with β-glycerophosphate (BG), BG with AA, or BG with AA and Iox2, a PHD2-specific inhibitor, DNA was extracted from MC3T3-E1 pre-osteoblasts and primary rib chondrocytes. An antibody specific for 5-hmC was used to pull down DNA rich in 5-hmC, and real-time PCR with primers for CpG-rich areas of osteoblast and chondrocyte gene promoters was used to determine the 5-hmC content of those promoters as an indirect measure of their demethylation status. (A) AA treatment significantly increased 5-hmC levels in the promoters of osteoblast differentiation genes alkaline phosphatase (ALP), Indian hedgehog (Ihh), and osterix (Osx). (B) Inhibition of PHD2 with Iox2 significantly decreased 5-hmC levels in the promoters of chondrocyte genes HIF1α and VEGF. Results are presented as mean fold-change ± SEM. *P < 0.05 (Student’s *t*-test), n = 3 per group.

Three-day treatment of primary and cell line osteoblasts and chondrocytes with AA led to increased levels of 5-hmC, a potential marker of DNA demethylation, as measured by dot blot (30–90% increases, *P* < 0.05; Fig 7A and 7B). This result was confirmed using ELISA with an antibody specific for 5-hmC: AA treatment increased levels of 5-hmC (160–790% increases, *P* < 0.05; Fig 7C). To determine whether AA-induced increases in 5-hmC occurred in genes involved in osteoblast differentiation, we used a 5-hmC antibody to pull down DNA rich in 5-hmC and ran real-time PCR with primers specific for CpG-rich regions of promoters of interest. We found that AA treatment increased levels of 5-hmC in the promoter regions of osteoblast differentiation genes *Alp*, *Ihh*, and *Osx* (1.6–2.4-fold increases, *P* < 0.05; Fig 8A). In order to determine whether PHD2 could be involved in this increase in 5-hmC, we treated ATDC5 chondrocytes with AA and Iox2, a PHD2-specific inhibitor. We found that inhibition of PHD2 significantly reduced 5-hmC content in the promoter regions of chondrocyte differentiation genes *Hif1α* and *Vegf* (0.2–0.4-fold decreases, *P* < *0.05*; Fig 8B).

**Fig 9:**
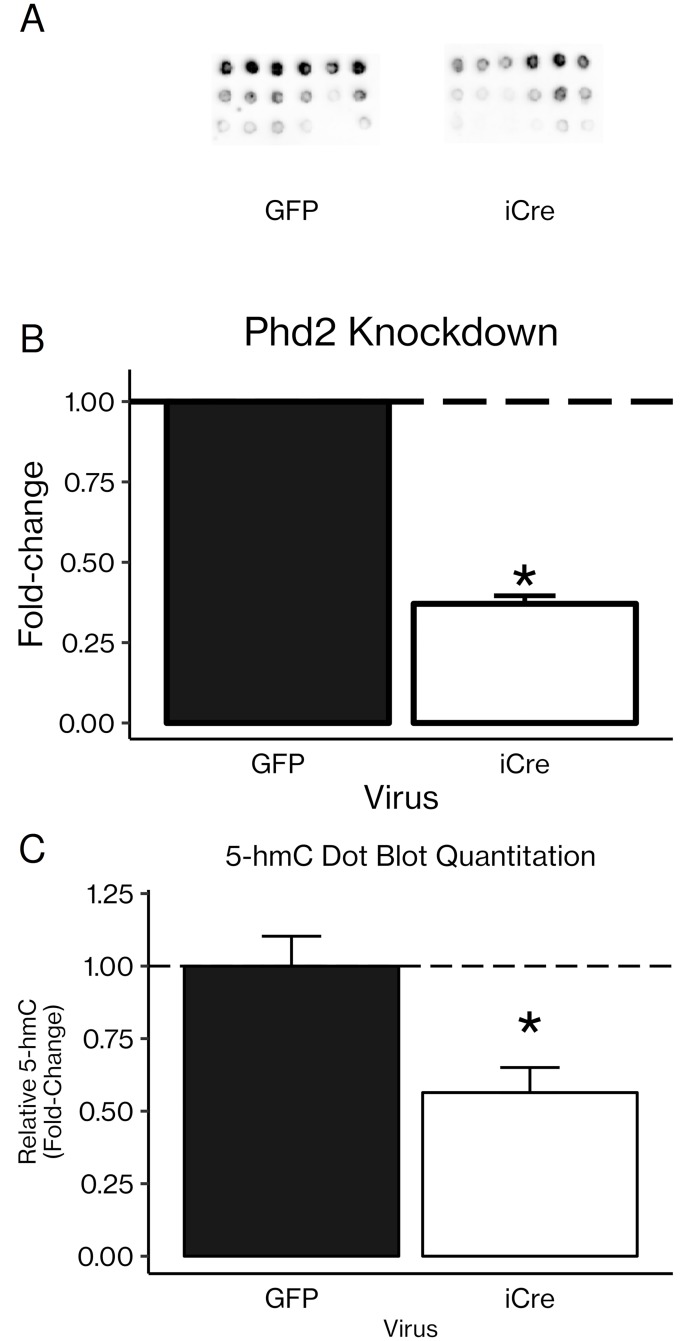
Knockdown of PHD2 significantly reduced 5-hmC levels in primary rib chondrocytes. (A) Primary rib chondrocytes from *Phd2*-floxed mice were transduced with an adenoviral vector carrying iCre or GFP as a control. DNA was extracted and dot blotted as in Fig 7. (B) *Phd2* gene expression after *Phd2*-floxed cells were transduced with adenoviral iCre. *P < 0.01 vs. adenoviral GFP (Student’s *t*-test), n = 3 per group. (C) Dot blots were quantitated with ImageJ. NB: The images shown in (A) are several exposures stacked to make differences more visible to the eye, while the quantitations in (C) were performed on single, non-stacked images. Results are presented as mean fold-change ± SEM. *P < 0.05 (Student’s *t*-test), n = 6 per group.

To further confirm whether PHD2 could itself mediate changes in 5-hmC levels, we used an adenoviral iCre vector to knock down *Phd2* in rib chondrocytes from *Phd2*-floxed mice (60% knockdown, *P* < 0.001). Knocking down *Phd2* led to a 0.56-fold decrease in 5-hmC (*P* < 0.05; Fig 9).

We and others have shown that vitamin C-mediated activation of PHD2 destabilizes HIF1α via proline hydroxylation-mediated proteasomal degradation. [26] Since HIF1α is a known regulator of Jumonji family demethylases, we evaluated whether AA regulates expression of Jumonji demethylases in osteoblasts or chondrocytes. Serum-free cultures of ATDC5 chondrocytes and MC3T3-E1 pre-osteoblasts were treated with vitamin C or vehicle, and RNA was extracted and used for real-time RT-PCR. We found expression of neither *Jmjd1a* nor *Jmjd2b* was affected by AA treatment in these cells (Fig 4E).

## Discussion

The association between vitamin C and human health has a long history. Diseases resulting from vitamin C deficiencies have been observed as far back as 1500 BC, and one of the first documented clinical trials in the 18^th^ century involved treating scurvy, a debilitating condition with musculoskeletal symptoms caused by a lack of vitamin C, with AA-rich citrus fruits. [23] More recently, epidemiological studies have examined the relationship between AA and bone health. Several cross-sectional studies have observed positive correlations between dietary vitamin C intake and bone mineral density (BMD) in postmenopausal women, [41–44] although in other studies this correlation was not significant, [45–48] and one study actually reported a significant negative correlation. [49] These inconsistencies are likely due to differences in study design and measurement techniques, however, and the fact the that the majority of studies have reported either significant positive correlations or positive but not significant correlations between vitamin C and skeletal outcomes suggest a likely complex but beneficial effect of vitamin C on bone. [23]

Similarly, several mouse or rat models have demonstrated the importance of AA for the skeleton. Unlike humans, who require dietary vitamin C, many animals including rodents are able to synthesize their own vitamin C. The skeletal phenotypes of several transgenic rodent models with mutations in the AA biosynthesis pathway and which are therefore AA-deficient have been analyzed. Vitamin C deficiency in these transgenic rodents has resulted in spontaneous fractures, missing trabeculae, and reduced cortical zones. [15–22] The fact that these skeletal phenotypes can be rescued by dietary supplementation with vitamin C suggests that it is vitamin C deficiency itself, not a secondary effect of the various mutations, which results in decreased BMD, trabecular number, and cortical thickness. Furthermore, these effects seem to be primarily due to decreased bone formation resulting from defective osteoblast function in AA-deficient animals. [23] Thus, vitamin C is an important regulator of bone health.

Consistent with these clinical observations, our findings show that AA-deficient *sfx* mice had decreased articular cartilage (Fig 1) and trabecular bone (Fig 2). However, the mechanisms for these changes have yet to be fully elucidated. In fact, our results suggest that vitamin C may have different effects in osteoblasts and chondrocytes. At the cellular level, AA is known to affect both chondrocytes and osteoblasts. In chondrocytes, AA has been shown to act via ERK signaling to increase chondrocyte proliferation and expression of chondrocyte differentiation genes. [12–14] Similarly, many studies demonstrate the ability of AA to stimulate osteoblast proliferation and differentiation, [3–11] and several of these studies implicate collagen synthesis as a mediator of these effects.

In this current study, we found that while *in vitro* AA treatment increased chondrocyte proliferation, AA had no significant effect on osteoblast proliferation (Fig 3A–3C). Conversely, AA had no effect on ALP activity in chondrocytes but significantly increased ALP activity in osteoblasts (Fig 3D–3F). The lack of significant effects of AA on proliferation in osteoblasts and differentiation in chondrocytes, which is in contrast to the findings of previous studies cited above, may be due to differences in the stage of cell maturation as well as treatment conditions and duration. Nevertheless, these results suggest that the primary effect of AA in chondrocytes is to maintain them in an immature, proliferative state while the primary effect of AA in osteoblasts is to induce differentiation.

Our mRNA expression data further support this explanation of the effects of AA on chondrocytes vs. osteoblasts. In chondrocytes, AA treatment increased expression of immature chondrocyte markers while tending to decrease expression of a marker of chondrocyte maturity (Fig 4A). In osteoblasts, AA treatment led to significant increases in markers of osteoblast differentiation (Fig 4B). Both of these effects would support the observed bone and cartilage phenotypes of *sfx* mice; the decrease in trabecular bone results from a lack of differentiated osteoblasts in the AA-deficient mice, and the decrease in articular cartilage results from a defect in chondrocyte differentiation and/or proliferation, potentially involving a lack of immature, proliferative chondrocytes to replace the cartilage as it is worn away in the AA-deficient mice.

In terms of molecular mechanisms of vitamin C actions, AA is a well-known antioxidant, and we have previously demonstrated the ability of AA to induce osteoblast differentiation by increasing binding of nuclear factor-E2-related factor-1 (Nrf1) to antioxidant response elements (AREs) in the osterix promoter. [24, 39] Both Nrf2 signaling and the induction of endoplasmic reticulum stress have been suggested as additional mechanisms of AA’s action. However, we did not find any significant changes in ER stress or Nrf genes in AA-treated ATDC5 chondrocytes (Fig 4C and 4D), suggesting that these are not primary mechanisms of AA’s action in these cells. An additional well established biological role of AA is as a cofactor for prolyl and lysyl hydroxylases. Hydroxylation of specific proline and lysine residues within the collagen molecule is required for collagen cross-linking, a crucial step in the collagen maturation process which contributes to building bone and cartilage. [3, 23, 50, 51] In addition to the regulation of collagen cross-linking, however, proline hydroxylation is known to play a role in regulation of other intracellular signaling pathways. AA is a cofactor for prolyl hydroxylase domain-containing (PHD) proteins, a family of dioxygenases which depend upon the presence of 2-oxoglutarate, Fe^2+^, and oxygen to function. [32] PHDs can hydroxylate proline residues in the hypoxia-inducible factors (HIFs), causing the HIFs to undergo ubiquitination followed by proteasomal degradation. [52] Indeed, we have demonstrated that PHD2-mediated proteasomal degradation of HIF1α is a mechanism of AA’s action in bone. [26]

Since AA is known to increase 5-hmC levels in DNA via the TETs, members of the same enzyme family as the PHDs, we decided to investigate TET- and/or PHD2-mediated increases in 5-hmC in osteoblasts and chondrocytes as a potential epigenetic mechanism for AA’s effects. Indeed, changes in 5-hmC levels have been reported in osteoarthritic human chondrocytes, [30] and hypomethylation of CpG islands in promoter regions of chondrocyte differentiation genes was observed in human synovium-derived mesenchymal stem cells, [31] suggesting that regulation of DNA demethylation may be important for musculoskeletal function. 5-hmC levels have been reported to rise in ATDC5 chondrocytic cells during differentiation, an effect mediated by TET1. [53] We confirmed that all three TET isoforms and all three PHD isoforms were expressed in both osteoblasts and chondrocytes at the mRNA level (Fig 5). The effect of AA treatment on TET and PHD expression varied by isoform, although TETs tended to increase while PHDs tended to decrease. However, the change in TET or PHD mRNA expression after AA treatment may be less important given that basal expression, of PHD2 at least, is quite high, and we are more interested in enzyme activity than changes in expression levels.

Furthermore, PHD2 is localized in both the cytoplasm and the nucleus, and AA treatment tended to increase the nuclear-to-cytoplasmic ratio of PHD2 expression (Fig 6). These findings confirm those of previous studies which have found that PHD2 is localized in both the cytoplasm and the nucleus and that PHD2 has significant hydroxylase activity in the nucleus. [54–57] Thus, PHD2 is present in the nucleus and could plausibly interact directly with DNA, contributing directly to AA-induced increases in DNA 5-hmC content, although this novel hypothesis remains to be investigated. Consistent with this possibility, our results show that AA increased 5-hmC levels in both osteoblasts and chondrocytes as measured by dot blot and ELISA (Fig 7). Since 5-hmC is the first product in the process of demethylating 5-mC, it is a possible indicator of DNA demethylation, although 5-hmC can exist as a stable epigenetic marker by itself.

Once we had confirmed that AA induced demethylation, we used a kit to pull down DNA rich in 5-hmC and then used real-time PCR with primers specific for CpG-rich regions of osteoblast gene promoters. This method gives us an indirect measure of how much the 5-hmC levels in those gene promoters have changed. We found significant increases in 5-hmC levels in osteoblast-specific genes *Alp*, *Ihh*, and *Osx* (Fig 8A), confirming that the global effect on 5-hmC does include osteoblast genes and supporting the hypothesis that some of AA’s effect in osteoblasts may in fact be due to increases in 5-hmC levels. Furthermore, inhibition of PHD2 using Iox2, a PHD2-specific inhibitor, resulted in significant decreases in 5-hmC in chondrocyte-specific genes *Hif1α* and *Vegf* (Fig 8B). In order to lend support to the hypothesis that the Iox2 effect on 5-hmC is mediated specifically via PHD2, we knocked down expression of the *Phd2* gene in rib chondrocytes isolated from *Phd2*-floxed mice *in vitro* using an adenoviral iCre vector. Knockdown of *Phd2* expression led to significantly decreased 5-hmC in the DNA isolated from these cells (Fig 9), suggesting for the first time that PHD2 may itself contribute to increased DNA 5-hmC content. However, there is not yet any evidence that PHD2 can directly modify DNA, and further mechanistic studies are needed to determine whether DNA can act as a substrate for PHD2.

While our findings provide evidence for the possibility of an epigenetic mechanism for AA’s effects in osteoblasts and chondrocytes, more work is needed to elucidate the specific TETs and/or PHDs which contribute to these changes in DNA 5-hmC content—particularly, to confirm the ability of PHD2 to directly increase 5-hmC levels—in addition to determining whether these changes in 5-hmC represent DNA demethylation and the extent to which this epigenetic mechanism contributes to AA effects in bone and cartilage *in vivo*. In particular, since PHD2 is known to mediate AA effects in bone and cartilage, and since we have reported here that knocking down *Phd2* expression decreased levels of 5-hmC in rib chondrocytes, our future studies will investigate the novel hypothesis that PHD2 may be directly involved in DNA demethylation. Additionally, methylation sequencing and pathway analysis could provide a much deeper understanding of which genes are epigenetically regulated by AA and lead to novel targets for future therapies. Thus, we are planning a series of methylation sequencing studies to determine the specific effects of AA deficiency and PHD2 ablation on chondrocyte and osteoblast epigenomes. Further understanding of the mechanisms of bone and cartilage development and maintenance is crucial for the development of novel therapeutic approaches for diseases such as osteoporosis and osteoarthritis, and this epigenetic mechanism of DNA 5-hmC regulation and demethylation is an excellent candidate pathway for the discovery of new druggable targets.

## Supporting information

S1 Fig AA increased differentiation of MC3T3-E1 cells *in vitro*. Proliferation of (A) ATDC5 chondrocytic cells under the specific conditions we observed and of (B) MC3T3-E1 osteoblasts was not changed by AA treatment. ALP activity of (C) ATDC5 chondrocytic cells under the specific conditions we observed was not changed by AA treatment. However, ALP activity of (D) MC3T3-E1 osteoblasts was increased by AA treatment. Results are presented as mean ± SEM. *P < 0.05 vs. control (pairwise *t*-tests with Bonferroni corrections for multiple comparisons [A,C,D] or Student’s *t*-test [B]), n = 6–8 per group.
(TIF)

S1 Table Ct and dCt (adjusted to housekeeping gene PPIA) for real-time RT-PCR of each PHD and TET isoform. (PDF)
