# Supplementary material for: Vitamin C effects on 5-hydroxymethylcytosine and gene expression in osteoblasts and chondrocytes: Potential involvement of PHD2
Source: PLoS One. 2019 Aug 7;14(8):e0220653. doi: 10.1371/journal.pone.0220653 (PMC6685624; doi:10.1371/journal.pone.0220653)
Supplement: S1 Table — (PDF) [file pone.0220653.s002.pdf]

Cell Type	Gene	Isoform	Treatment	Ct.Mean	Ct.SEM	dCt.Mean	dCt.SEM
Articular chondrocytes	PHD	1	BG	19.74	0.2325	4.938	0.1877
Articular chondrocytes	PHD	1	AA	19.53	0.1127	4.608	0.1188
Articular chondrocytes	PHD	2	BG	19.21	0.2173	4.535	0.2081
Articular chondrocytes	PHD	2	AA	19.51	0.0591	4.665	0.0548
Articular chondrocytes	PHD	3	BG	25.54	0.1336	10.788	0.1434
Articular chondrocytes	PHD	3	AA	26.24	0.0878	11.321	0.0820
Articular chondrocytes	TET	1	BG	28.88	0.6478	14.082	0.6061
Articular chondrocytes	TET	1	AA	27.27	0.2453	12.348	0.2483
Articular chondrocytes	TET	2	BG	24.41	0.1022	9.659	0.1009
Articular chondrocytes	TET	2	AA	24.10	0.1158	9.181	0.1195
Articular chondrocytes	TET	3	BG	25.25	0.6662	10.444	0.6302
Articular chondrocytes	TET	3	AA	23.42	0.1728	8.502	0.1789
ATDC5	PHD	1	BG	19.36	0.1732	4.728	0.1261
ATDC5	PHD	1	AA	19.34	0.2601	4.627	0.1048
ATDC5	PHD	2	BG	18.48	0.1000	3.847	0.0668
ATDC5	PHD	2	AA	19.45	1.0770	4.740	1.0081
ATDC5	PHD	3	BG	25.64	0.0560	11.004	0.0607
ATDC5	PHD	3	AA	25.61	0.2372	10.900	0.0959
ATDC5	TET	1	BG	23.14	0.2029	8.506	0.1655
ATDC5	TET	1	AA	23.09	0.3860	8.385	0.2298
ATDC5	TET	2	BG	23.11	0.1157	8.476	0.0966
ATDC5	TET	2	AA	22.89	0.4620	8.182	0.3062
ATDC5	TET	3	BG	20.65	0.2851	6.020	0.2422
ATDC5	TET	3	AA	20.74	0.2329	6.036	0.2469
Calvarial osteoblasts	PHD	1	BG	19.88	0.0757	5.149	0.0679
Calvarial osteoblasts	PHD	1	AA	20.17	0.0528	5.456	0.0665
Calvarial osteoblasts	PHD	2	BG	17.86	0.1024	3.130	0.0844
Calvarial osteoblasts	PHD	2	AA	19.34	0.0647	4.626	0.0832
Calvarial osteoblasts	PHD	3	BG	22.85	0.0255	8.117	0.0239
Calvarial osteoblasts	PHD	3	AA	24.80	0.2704	10.089	0.2889
Calvarial osteoblasts	TET	1	BG	27.88	0.1355	13.156	0.1353
Calvarial osteoblasts	TET	1	AA	27.96	0.2670	13.252	0.2783
Calvarial osteoblasts	TET	2	BG	23.28	0.1511	8.549	0.1325
Calvarial osteoblasts	TET	2	AA	23.55	0.0817	8.844	0.0980
Calvarial osteoblasts	TET	3	BG	23.00	0.1515	8.275	0.1531
Calvarial osteoblasts	TET	3	AA	23.62	0.5788	8.906	0.6005
MC3T3-E1	PHD	1	BG	19.78	0.2631	5.304	0.2353
MC3T3-E1	PHD	1	AA	19.56	0.0677	5.059	0.0603
MC3T3-E1	PHD	2	BG	18.24	0.2186	3.756	0.1912
MC3T3-E1	PHD	2	AA	18.02	0.0598	3.521	0.0523
MC3T3-E1	PHD	3	BG	26.04	0.0873	11.595	0.1107
MC3T3-E1	PHD	3	AA	25.75	0.0685	11.249	0.0613
MC3T3-E1	TET	1	BG	25.47	0.1637	11.021	0.1877
MC3T3-E1	TET	1	AA	24.67	0.0817	10.174	0.0741
MC3T3-E1	TET	2	BG	22.76	0.2272	8.316	0.2518
MC3T3-E1	TET	2	AA	22.44	0.0802	7.940	0.0998
MC3T3-E1	TET	3	BG	21.83	0.2009	7.386	0.2259
MC3T3-E1	TET	3	AA	21.30	0.0892	6.806	0.0877
Rib chondrocytes	PHD	1	BG	19.78	0.0475	4.938	0.0722
Rib chondrocytes	PHD	1	AA	19.72	0.0650	4.700	0.0484
Rib chondrocytes	PHD	2	BG	17.83	0.0260	2.992	0.0374
Rib chondrocytes	PHD	2	AA	19.30	0.0724	4.279	0.0947
Rib chondrocytes	PHD	3	BG	20.48	0.0341	5.647	0.0599
Rib chondrocytes	PHD	3	AA	23.84	0.1431	8.821	0.1608
Rib chondrocytes	TET	1	BG	27.07	0.1075	12.230	0.0971
Rib chondrocytes	TET	1	AA	27.46	0.0485	12.445	0.0511
Rib chondrocytes	TET	2	BG	22.81	0.0687	7.976	0.0903
Rib chondrocytes	TET	2	AA	23.43	0.0136	8.383	0.0765
Rib chondrocytes	TET	3	BG	22.64	0.1246	7.806	0.1380
Rib chondrocytes	TET	3	AA	23.25	0.3406	8.232	0.3401
